# Evaluation of model-based contouring versus 2D segmentation for cardiac mass and volumes

**DOI:** 10.1186/1532-429X-11-S1-P219

**Published:** 2009-01-28

**Authors:** Andrew S Chi, Cuilian Miao, Chia-Ying Liu, Patricia A Cleary, Joao AC Lima, David A Bluemke

**Affiliations:** 1 Johns Hopkins, Baltimore, MD USA; 2 The George Washington University, Rockville, MD USA

**Keywords:** Left Ventricular Mass, Myocardial Mass, Peak Filling Rate, Peak Ejection Rate, SSFP Cine

## Objective

To compare three-dimensional, model-based contouring versus two-dimensional, semi-automatic contouring of cardiac cine MRI for determination of left ventricular mass and volumes.

## Background

Traditional cardiac MRI methods have relied on slice-by-slice contouring and mathematical calculations to estimate myocardial mass and volumes. Three-dimensional model-based algorithms offer the potential advantages of faster analysis times, increased reproducibility, and physiologically-relevant information that includes the complex motion of the mitral valve, balanced by potential sensitivity to slice misregistration related to multiple breath-holds.

## Methods and results

100 cardiac MRI SSFP cine studies were evaluated using a model-based software (Cardiac Image Modeller (CIM)) and a semiautomatic contouring system (MASS). Parameters of cardiac function included left ventricular (LV) end diastolic mass (EDM), end diastolic volume (EDV), end systolic volume (ESV), and ejection fraction (EF). Peak ejection rate (PER) and peak filling rate (PFR) were also included. Reproducibility data for intra-observer variability was performed for 20 consecutive cases.

## Results

**Figure 1:**
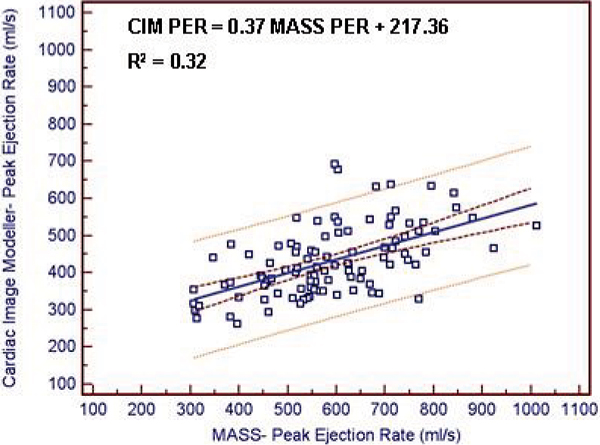
Figure 1

**Figure 2:**
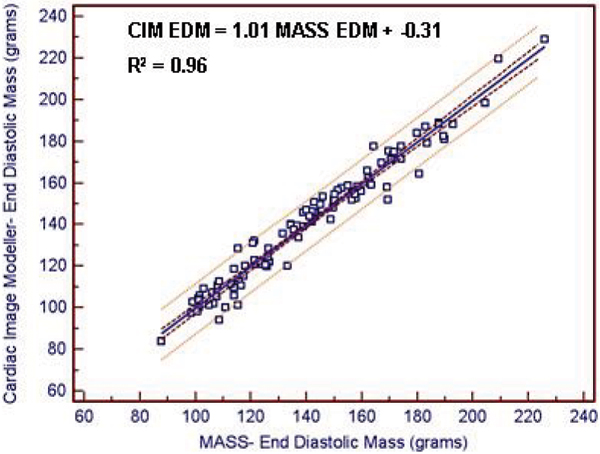
Figure 2

Bland Altman analysis comparing CIM and MASS software demonstrated a high degree of agreement for left ventricular mass and volumes. Mean differences (± limits of disagreement defined as ± 2 SD) were -0.2 g (-11.8–11.5 g) for EDM, 0.1 ml (-8.5–8.6 ml) for EDV, -0.3 ml (-6.0–5.4 ml) for ESV, and 0.3% (-3.6–4.2%) for EF. The relationship between CIM and MASS was linear (Figure 1) and highly correlated for left ventricular end diastolic mass (R^2^ = 0.96) and volumes (R^2^ = 0.98). Intra-observer mean differences (± 2 SD) using the CIM software were -2.5 g (-13.2–8.2 g) for EDM, 3.2 ml (-10.4–16.7 ml) for EDV, 1.6 ml (-4.7–8.0 ml) for ESV and -0.4% (-5.4–4.6%) for EF. Intraclass correlation coefficients for left ventricular EDM, EDV, ESV, and EF were 0.99 (0.988–0.998), 0.99 (0.97–0.99), 0.99 (0.98–1.0), and 0.96(0.90–0.99), respectively. For filling and emptying rates (Figure 2), large variations between the two methods were identified. Mean differences (± 2 SD) were -155.2 ml/s (-388.5–78.1 ml/s) for PER and -109.7 ml/s (-357.9–138.4 ml/s) for PFR.

## Conclusion

Compared to two-dimensional contouring techniques for cardiac MRI analysis, model-based contouring provides a reliable alternative for quantitative determination of myocardial mass and volumes. Characterization of systolic emptying and diastolic filling rates varies significantly between the two methods; further study is needed to understand these differences.

